# Development of an integrated Sasang constitution diagnosis method using face, body shape, voice, and questionnaire information

**DOI:** 10.1186/1472-6882-12-85

**Published:** 2012-07-04

**Authors:** Jun-Hyeong Do, Eunsu Jang, Boncho Ku, Jun-Su Jang, Honggie Kim, Jong Yeol Kim

**Affiliations:** 1 Constitutional Medicine & Diagnosis Research Group, Medical Research Division, Korea Institute of Oriental Medicine, 1672 Yuseongdae-ro, Yuseong-gu, Daejeon 305-811, Republic of Korea; 2 Department of Information and Statistic, Chungnam National University, 79 Daehang-ro, Yuseong-gu, Daejeon, 305-764, Republic of Korea

**Keywords:** Sasang constitution, Diagnosis, Face, Body shape, Voice, Questionnaire, Integration

## Abstract

**Background:**

Sasang constitutional medicine (SCM) is a unique form of traditional Korean medicine that divides human beings into four constitutional types (Tae-Yang: TY, Tae-Eum: TE, So-Yang: SY, and So-Eum: SE), which differ in inherited characteristics, such as external appearance, personality traits, susceptibility to particular diseases, drug responses, and equilibrium among internal organ functions. According to SCM, herbs that belong to a certain constitution cannot be used in patients with other constitutions; otherwise, this practice may result in no effect or in an adverse effect. Thus, the diagnosis of SC type is the most crucial step in SCM practice. The diagnosis, however, tends to be subjective due to a lack of quantitative standards for SC diagnosis.

**Methods:**

We have attempted to make the diagnosis method as objective as possible by basing it on an analysis of quantitative data from various Oriental medical clinics. Four individual diagnostic models were developed with multinomial logistic regression based on face, body shape, voice, and questionnaire responses. Inspired by SCM practitioners’ holistic diagnostic processes, an integrated diagnostic model was then proposed by combining the four individual models.

**Results:**

The diagnostic accuracies in the test set, after the four individual models had been integrated into a single model, improved to 64.0% and 55.2% in the male and female patient groups, respectively. Using a cut-off value for the integrated SC score, such as 1.6, the accuracies increased by 14.7% in male patients and by 4.6% in female patients, which showed that a higher integrated SC score corresponded to a higher diagnostic accuracy.

**Conclusions:**

This study represents the first trial of integrating the objectification of SC diagnosis based on quantitative data and SCM practitioners’ holistic diagnostic processes. Although the diagnostic accuracy was not great, it is noted that the proposed diagnostic model represents common rules among practitioners who have various points of view. Our results are expected to contribute as a desirable research guide for objective diagnosis in traditional medicine, as well as to contribute to the precise diagnosis of SC types in an objective manner in clinical practice.

## Background

Sasang constitutional medicine (SCM) is a unique form of traditional Korean medicine that divides human beings into four constitutional types (Tae-Yang: TY, Tae-Eum: TE, So-Yang: SY, and So-Eum: SE), which differ in inherited characteristics, such as external appearance, personality traits, susceptibility to particular diseases, drug responses, and equilibrium among internal organ functions [1]. In contrast to the diagnostic procedure of traditional Chinese medicine, which places the greatest importance on the ‘syndrome,’ SCM places emphasis on the ‘constitution,’ and the therapeutic decision is then mostly based on which SC type the patient is [2]. According to SCM, each constitutional type corresponds to a certain group of medicinal herbs and herbal remedies. This relationship means that herbs that belong to a certain constitution cannot be used in patients with other constitutions; otherwise, this practice may result in no effect or in an adverse effect. Thus, the diagnosis of SC type is the most crucial step in SCM practice [3].

In agreement with other forms of complementary and alternative medicine [4-7], the diagnostic process in SCM remains subjective and unreliable due to a lack of quantitative standards for SC diagnosis [8]. So far, attempts at standardization have focused on facial, body shape, voice analyses, and questionnaires. In terms of facial analysis, facial metrics on 2D and 3D images have been employed to investigate the typical features of SC types and to develop predictive models for SC types [9-13]. In body shape analysis, five horizontal lines [14,15] and eight circumferences [16,17] of the trunk are commonly used. Research on voice analysis has been undertaken regarding various vocal features, such as the pitch, frequency, reading speed, shimmer, harmonics, formants, and energy of the voice, using computerized speech laboratory methods [18-20]. In 2004, a voice analysis system named the Phonetic System for Sasang Constitution-2004 was successfully developed [21,22]. Questionnaires have been used in the earliest trials of SC diagnosis [23,24]. Today, the Questionnaire for Sasang Constitutional Classification II (QSCCII) is a commonly used questionnaire with qualitative components concerning face, body shape, voice, and personal characteristics, in addition to physiological symptoms [25].

Most of this research has focused on the construction of individual diagnostic models using data from a small number of clinical sites, which was insufficient to guarantee the validity of the diagnostic models. In clinical practice, SCM practitioners employ all four examinations to determine patients’ SC types. This holistic approach provides better information about the patients’ constitutions and ailments, which cannot be obtained by individual examination. An integrated diagnostic tool that simulates SCM practitioners’ holistic diagnostic processes would be a more desirable approach.

In this study, four standardized individual diagnostic models were developed based on a large amount of data that were acquired from various Oriental medical clinics for the four diagnostic components: face, body shape, voice, and questionnaire responses. For a quantitative and objective analysis, engineering techniques and statistical data analysis including multinomial logistic regression were employed. Inspired by SCM practitioners’ holistic diagnostic processes, an integrated diagnostic model was then proposed by combining the four individual models. The validity of the proposed model was confirmed by applying it to a test set that was not used in developing the model.

## Methods

### Participants and data acquisition

We strictly controlled the characteristics of the practitioners and subjects. 23 and more SCM practitioners, who had more than five years of experience in clinical practice, diagnosed the patients’ SC types. Their SC types were confirmed by observing improvements after the administration of constitution-specific pharmaceuticals over one month. A more detailed procedure of determining SC type is described in Song et al. [26].

We collected face, body shape, voice, and questionnaire data from the subjects using a standard operating procedure that was developed for the Korea Constitutional Multicenter Study [27]. The collected data included photographs with neutral expressions from the frontal and profile points of view; height, weight, and eight body shape circumferences; voice signal recordings of five vowels (‘a,’ ‘e,’ ‘i,’ ‘o,’ and ‘u’) and two repeated sentences; and questionnaires for yin/yang characteristics and physiological symptoms. This process was approved by the Korea Institute of Oriental Medicine (KIOM) - Institutional Review Board (I-0910/02-001) and we obtained written informed consent from the subjects.

From 23 sites (Oriental medical clinics), 2,973 patients, ranging in age from teenagers to people in their eighties, were recruited between November of 2007 and July of 2011 (see Additional file 1). Among these 2,973 patients, 2,462 patients recruited between November of 2007 and July of 2010 were assigned to training set for the construction of a diagnostic model, whereas the others were assigned to test set for the validation of the constructed model. All data that contained clinical information were stored in the Korea Constitutional Multicenter Bank at KIOM.

Several patients were excluded for various reasons. TY-type subjects were unavoidably excluded due to the small sample size of the TY type. Subjects younger than 15 years of age were also excluded on the basis that the phenotypic characteristics of these subjects would greatly fluctuate based on age during the adolescent period. And improper data were excluded from each component. Table 1 summarizes the excluded data and the number of used data for the training and test of each model after the exclusion.

**Table 1:** The numbers of excluded and used data for the training and test of each individual diagnostic model

**List of exclusion reasons (Total no. of subjects: training 2462; test 511)**	**Training**				**Test**			
	**Face**	**Body shape**	**Voice**	**Questionnaire**	**Face**	**Body shape**	**Voice**	**Questionnaire**
Initially not collected	22	0	409	409	0	0	0	0
Low quality pictures or voice recording files	133	-	132	-	6	-	14	-
TY samples	58	59	54	56	6	6	6	6
Subjects younger than 15 years old	52	54	39	41	13	13	11	13
Data extraction errors and missing cases	1189	97	3	0	132	0	0	0
Outliers considered in the calculation of the moving average	46	27	86	16	0	7	26	0
Excluding influential samples	6	11	49	25	0	0	0	0
Final no. of subjects	956	2214	1687	1915	354	485	480	492
Common samples after integrating the four components (for the integrated diagnostic model)	729 (male: 241, female: 488)				346 (male: 114, female: 232)			

### Candidate feature variables

#### Facial images

Candidate feature variables expressing facial characteristics were created with facial points and contours, which were automatically extracted via image processing techniques. The positions of the numbered facial points and features are shown in Additional file 2, and the candidate feature variables are described in Additional file 3.

#### Body shape

The eight circumferences of body shape, ratios of all possible pairs of eight circumferences, height, weight, and body mass index (BMI) were used as candidate variables expressing body shape characteristics. The measurement methods of the circumferences are described in Additional file 4, where a standardized tapeline (150 cm/60 inches, Hoechstmass, Germany) was used to measure body shape.

#### Voice

Voice features were extracted using two voice analysis programs, HTK [28] and Praat [29]. The size of a window, i.e., the minimum duration of a voice signal for feature extraction, was 40 ms, and neighboring windows overlapped by 50%. We used 41 features for each vowel and 17 features for the sentences. The descriptions of vowel and sentence features are shown in Additional file 5 and 6, respectively. A total of 222 features were extracted as an initial feature set. Next, we applied a genetic algorithm-based feature selection technique using Weka [30] to reduce the total number of features. Finally, 88 features were selected and used in diagnostic model learning.

#### Questionnaire

Binary variables representing personality characteristics and physiological symptoms were constructed using the response categories of the questions in the questionnaire, which consisted of 67 multiple-choice questions (see Additional file 7). Because face, body shape, and voice features were all represented by continuous variables, efforts were made to generate continuous variables using the binary variables that were obtained with the questionnaire so as to apply the same analytic method used for the other features. The procedure of generating continuous variables is summarized in Additional file 8[31]. Selected significant binary variables are also shown in Additional files 9, 10, 11, 12, 13 and 14.

#### Compensating for age differences

Because the candidate feature variables may have shown age-specific trends, a process to eliminate the effect of age was considered. To estimate the non-linear trend of each feature variable according to age, moving averages and standard deviations of the variables at each age were calculated using the samples within an age range of ±5 years for the given age. In calculating the moving average, outliers were excluded based on the results of the multivariate outlier detection method [32,33]. Each candidate feature variable was then normalized with the average and the standard deviation of the variable.

### Model for Sasang constitution diagnosis

#### Individual diagnostic models

Before building the individual diagnostic model for each component, unusual samples were excluded that influenced the predicted estimates. Two binary logistic regression analyses that compared TE to SE and SE to SY were conducted, and the unusually influential samples were then identified by calculating Cook’s distance and standard residuals [34]. The excluded data are provided in Table 1.

With the data remaining after excluding the influential samples, individual diagnostic models were developed by multinomial logistic regression (MLR) based on stepwise forward variable selection using the score statistic and Wald’s test [35,36]. Each individual diagnostic model consisted of two independent models: one was for male patients and the other for female patients. The age factor was forced into each model as a baseline covariate for the adjustment. For the diagnostic model that was generated using questionnaire information, the factors of age, education and occupation level, which may have affected the questionnaire responses, were used as baseline covariates.

#### Integrating diagnostic models from four diagnostic components

Let *π*_*ij*_ be the estimated probability of the *i*^*th*^ subject in category *j* for each individual diagnostic model, where *j* = 1, 2, and 3 indicates the TE, SE, and SY types, respectively. The estimated probability can be regarded as a score for the different SC types, which we called SC score; hence, it was feasible to consider the total of each *π*_*ij*_ derived from the four individual diagnostic models as the integrated score. In addition, the importance of each individual diagnostic model was also considered by multiplying the weights by *π*_*ij*_. The integrated score of an SC type *j* for the *i*^*th*^ subject, denoted as *TSCORE*_*ij*_, can be defined by the sum of (*π*_*ij*_)_*r*_ with weight *w*_*r*_ :

(1) $$\text{TSCOR}E_{\mathrm{ij}}=\sum_{r=1}^{4}w_{r}{\left(\pi_{\mathrm{ij}}\right)}_{r}\text{,}$$

where *r* indicates each individual diagnostic component; *r *= 1, 2, 3, and 4, representing face, body shape, voice, and questionnaire, respectively.

**Figure 1:**
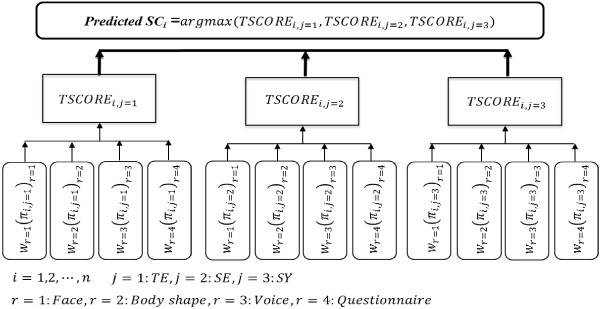
Schematic flow chart of the integration process using the probability scores from the four diagnostic components.

In this study, it was assumed that all individual diagnostic components equally contributed to the diagnosis of the SC type, and, hence, all weights for each individual SC score were initially set to 1. Finally, the predicted SC type for the *i*^*th*^ subject was determined by choosing the maximum value between *TSCORE*_*i*1_, *TSCORE*_*i*2_, and *TSCORE*_*i*3_. This process can be expressed as the following equation:

(2) $$\text{Predicted}\;SC_{i}=\text{argmax}\left(\text{TSCOR}E_{i1}\text{,}\;\text{TSCOR}E_{i2}\text{,}\;\text{TSCOR}E_{i3}\right)\text{,}\;$$

where each number of the subscriptions indicates TE, SE, and SY. For better clarity, the schematic diagnosis algorithm is illustrated in Figure 1.

## Results

### Predicted results of the individual diagnostic models

The accuracies of the created individual diagnostic models for both the training and test sets are shown in Table 2, and the selected variables and estimated parameters that were created by the MLR are summarized in Additional files 15, 16, 17, 18, 19, 20, 21 and 22.

**Table 2:** Accuracy of the individual diagnostic models

	**Male**				**Female**			
	**Face**	**Body shape**	**Voice**	**Questionnaire**	**Face**	**Body shape**	**Voice**	**Questionnaire**
Training Set	57.5%	61.3%	57.1%	57.4%	55.6%	54.2%	46.1%	53.8%
Test Set	60.5%	54.8%	39.9%	57.7%	42.9%	60.2%	37.5%	43.2%

The accuracies of the face, body shape, voice, and questionnaire models were 57.5%, 61.3%, 57.1%, and 57.4%, respectively, in the training set and were 60.5%, 54.8%, 39.9%, and 57.7%, respectively, in the test set for male patients. The accuracies of the face, body shape, voice, and questionnaire models were 55.6%, 54.2%, 46.1%, and 53.8%, respectively, in the training set and 42.9%, 60.2%, 37.5%, and 43.2%, respectively, in the test set for female patients.

The results indicate that the accuracies for male patients were higher than for female patients, except for the test results for body shape. In the diagnostic model for voice, the accuracy of the test set drastically decreased compared to that of the training set, which meant that the validity of the diagnostic model for voice was less than that of the other individual diagnostic models. In addition, the coefficient of determination *R*^2^ for the model for voice was relatively lower than the others (see Additional files 15, 16, 17, 18, 19, 20, 21 and 22).

### Predicted results of the integrated diagnostic model

The proposed integrated diagnostic model was tested with the lower weight for voice (*w*_3_ = 0.5), as well as with equal weights (*w*_r_ = 1), to examine the effect of reducing the weight for voice due to its low validity. Both trials, wherein an equal or half weight was established for voice, are evaluated in Tables 3 and 4, respectively.

**Table 3:** **Diagnostic results of the integrated diagnostic model with equal weights (*w***_***r***_ **= 1)**

	**Male**							**Female**						
			**Predicted SC type**							**Predicted SC type**				
			**TE**	**SE**	**SY**	**Total**	**Sensitivity**			**TE**	**SE**	**SY**	**Total**	**Sensitivity**
	Training set	True	TE	87	2	7	96	90.6%	True	TE	125	2	43	170	73.5%
	SC	SE	14	38	11	63	60.3%	SC	SE	16	50	60	126	39.7%	
	Type	SY	29	11	42	82	51.2%	type	SY	36	21	135	192	70.3%	
		Total	130	51	60	241			Total	177	73	238	488		
	Accuracy		69.3%					Accuracy		63.5%					
			**TE**	**SE**	**SY**	**Total**	**Sensitivity**			**TE**	**SE**	**SY**	**Total**	**Sensitivity**	
Test set	True	TE	47	2	5	54	87.0%	True	TE	55	4	35	94	58.5%	
	SC	SE	7	14	6	27	51.9%	SC	SE	5	27	39	71	38.0%	
	Type	SY	16	8	9	33	27.3%	type	SY	23	5	39	67	58.2%	
		Total	70	24	20	114			Total	83	36	113	232		
	Accuracy		61.4%					Accuracy		52.2%					

**Table 4:** **Diagnostic results of the integrated diagnostic model with the lower weight for voice (*w***_***3***_ **= 0.5)**

	**Male**							**Female**						
			**Predicted SC type**							**Predicted SC type**				
			**TE**	**SE**	**SY**	**Total**	**Sensitivity**			**TE**	**SE**	**SY**	**Total**	**Sensitivity**
Training set	True	TE	85	3	8	96	88.5%	True	TE	122	2	46	170	71.8%
	SC	SE	12	37	14	63	58.7%	SC	SE	16	51	59	126	40.5%
	Type	SY	27	11	44	82	53.7%	type	SY	38	23	131	192	68.2%
		Total	124	51	66	241			Total	176	76	236	488	
	Accuracy		68.9%					Accuracy		62.3%				
			**TE**	**SE**	**SY**	**Total**	**Sensitivity**			**TE**	**SE**	**SY**	**Total**	**Sensitivity**
Test set	True	TE	47	3	4	54	87.0%	True	TE	55	5	34	94	58.5%
	SC	SE	7	14	6	27	51.9%	SC	SE	3	31	37	71	43.7%
	type	SY	13	8	12	33	36.4%	type	SY	21	4	42	67	62.7%
		Total	67	25	22	114			Total	79	40	113	232	
	Accuracy		64.0%					Accuracy		55.2%				

Overall, the results of the integrated model were superior to those of the individual models. The accuracy of the integrated model with equal weights was 69.3% for the training set and 61.4% for the test set in male patients and 63.5% for the training set and 52.2% for the test set in female patients. The accuracy of the integrated model, applying *w*_3_ = 0.5, was 68.9% for the training set and 64.0% for the test set in male patients and 62.3% for the training set and 55.2% for the test set in female patients.

The result when applying *w*_3_ = 0.5 was more accurate than when applying *w*_3_ = 1 for the test set, whereas the accuracy of the model when applying *w*_3_ = 0.5 was lower than when applying *w*_3_ = 1 for the training set. This result indicated that the validity of the model when applying *w*_3_ = 0.5 was higher than when applying *w*_3_ = 1. The sensitivity of the model was relatively high for TE but was comparatively low for SY male and SE female.

**Figure 2:**
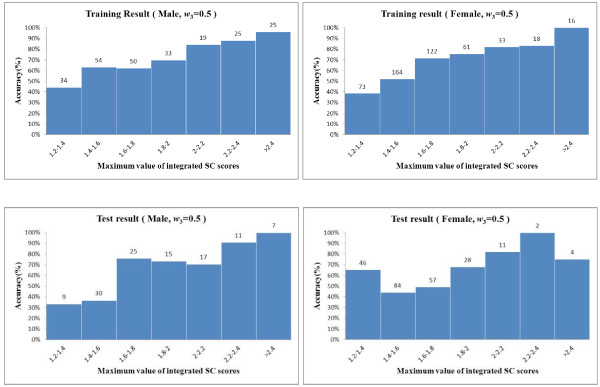
Accuracy of the integrated diagnostic model according to the maximum value of the integrated SC scores.

Figure 2 depicts the accuracy of the integrated model according to the maximum value of the integrated SC scores, *max*(*TSCORE*_*i*1_, *TSCORE*_*i*2_, *TSCORE*_*i*3_). Accuracy showed an increasing trend as the maximum value increased. To enhance the accuracy of the diagnostic model, a cut-off value was selected to diagnose only those subjects whose maximum value of integrated SC scores was higher than the cut-off value. If a cut-off value of 1.6 was chosen, for example, then the accuracies of the integrated model were 76.3% and 70.4% for male and female patients, respectively, in the training set (*w*_3_ = 0.5) and 78.7% and 59.8% for male and female patients, respectively, in the test set (*w*_3_ = 0.5).

## Discussion & conclusion

In this study, attempts were made to design a diagnostic model that was as objective as possible based on an analysis of quantitative data from various Oriental medical clinics. We tried to extract common criteria for SC diagnosis from a great variety of data, including practitioners’ varying points of view. Four individual diagnostic models for face, body shape, voice, and questionnaire information were obtained. Then, an integrated model was proposed that was inspired by SCM practitioners’ holistic diagnostic processes, which provided more sufficient information and which could be a more desirable approach for SC diagnosis.

In the individual training set, the diagnostic accuracy for body shape in the group of male patients was as high as 61.3%. The accuracies for face and questionnaire responses were almost the same, whereas that of voice was slightly lower. In the group of female patients, the diagnostic accuracy for face was 55.6%, followed by body shape, questionnaire, and voice, respectively, with almost an identical accuracy except for voice, as in the case of the male patients. The relatively low accuracy of the model for voice implies that it is difficult to find a stable diagnostic rule for voice. The individual diagnostic accuracies in the test set were slightly lower than those of the training set; however, the decrease in accuracy was within an acceptable range, except for the model for voice.

The diagnostic accuracies in the training set, after the four individual models had been integrated into a single model, improved to 69.3% and 63.5% in the male and female patient groups, respectively; however, when the model was applied to the test set, the accuracies decreased to 61.4% in the male patients and 52.2% in the female patients. The poor individual performance for voice was blamed, which led us to test an alternative integrated model by assigning a weight of half for voice. Although the new model showed almost the same diagnostic accuracy for the training set (68.9% in male patients and 62.3% in female patients), it clearly produced a greater accuracy in the test set (64.0% in male patients and 55.2% in female patients).

Although the resulting diagnostic power was not great, it was better than that of QSCCII [25], which is 51% and has been widely used for the diagnosis of SC type [37]. It may be difficult to directly compare our new method to QSCCII because QSCCII was developed using only questionnaire information and was tested on limited data collected from a single site; however, an individual’s response to qualitative questions on the four diagnostic components in the QSCCII could be subjective depending on his/her own point of view. It should be noted that the integrated model using quantitative data is superior in terms of validity and objectiveness.

A more desirable result was that a higher integrated SC score corresponded to a higher diagnostic accuracy, as shown in Figure 2. Using a cut-off value for the integrated SC score, such as 1.6, the accuracies increased by 14.7% in male patients and by 4.6% in female patients in the test set.

The insufficient diagnostic accuracies can be explained as follows. First, we collected data from 23 different sites, which incurred the possibility that the data might still contain too many practitioners' subjective opinions. This fact could have affected the SC diagnostic accuracy, although the characteristics of the practitioners and the subjects were kept very strict. Evidence of there being different opinions among practitioners can be found in other research [38,39].

Second, some of the variables that were extracted from face, body shape, voice, and questionnaire responses did not show clear differences among the SC types (see Additional files 9, 10, 11, 12, 13, 14, 15, 16, 17, 18, 19 and 20). This finding reveals that there still exists limitation to fully describing constitutional characteristics, as listed quantitatively in the SCM literature.

Third, because the Confucian culture of Korea has an influence on Korean women to modify their characteristics and their voices in talking, it is more difficult to obtain a diagnostic model in female patients than in male patients. The relatively low accuracies for questionnaire responses and voice partly explain this influence.

The proposed model was implemented in the form of a web-based prototype and is currently being tested in several clinics to get feedback from the practitioners. In the future, it will be necessary to collect more data on the TY type to complete the SC diagnostic model. Despite 2,973 samples being collected, only 1,075 samples were used, mainly due to a lack of featured extraction techniques and a lack of data quality control. It is necessary to develop an advanced technique for automatic feature extraction and to explore new feature variables for an improved diagnostic method. A different weighting method might be considered to properly reflect the importance of each diagnostic component. Furthermore, a future study may place emphasis on improving the performance for the groups which have poor diagnostic accuracies and sensitivities. At this point, we might need to analyze the typical subjects chosen by consensus from among SCM practitioners.

This study represents the first trial of integrating the objectification of SC diagnosis based on quantitative data and SCM practitioners’ holistic diagnostic processes. Although the diagnostic accuracy was not great, it is noted that the proposed diagnostic model represents common rules among practitioners who have various points of view. Our results are expected to contribute as a desirable research guide for objective diagnosis in traditional medicine, as well as to contribute to the precise diagnosis of SC types in an objective manner in clinical practice.

## Abbreviations

BMI: Body mass index; KIOM: Korea Institute of Oriental Medicine; MLR: Multinomial logistic regression; SC: Sasang constitution; SCM: Sasang constitutional medicine; SE: So-Eum; SY: So-Yang; TE: Tae-Eum; TY: Tae-Yang; QSCC: Questionnaire for the Sasang constitution classification.

## Competing interests

The authors hold and are currently applying for patents relating to the content of the manuscript.

## Authors’ contributions

JHD and EJ conceived the idea, designed the experiments, and interpreted the experimental results. BK, JSJ, and HK performed statistical analysis and interpretation of data. JYK designed the study and interpreted the experimental results. All authors contributed to manuscript preparations and approved the final manuscript.

## Pre-publication history

The pre-publication history for this paper can be accessed here:

http://www.biomedcentral.com/1472-6882/12/85/prepub

## Supplementary Material

Additional file 1 **Table S1. **Population characteristics of the participants. Click here for file

Additional file 2 **Figure S1. **Facial points used to calculated candidate feature variables. Click here for file

Additional file 3 **Table S2. **Feature variables expressing the facial characteristics. Click here for file

Additional file 4 **Table S3. **Measurement methods of eight circumferences for body shape. Click here for file

Additional file 5 **Table S4. **Description of vowel features. Click here for file

Additional file 6 **Table S5. **Description of sentence features. Click here for file

Additional file 7 **Table S6. **List of questions. Click here for file

Additional file 8 **Table S7. **The procedure of generating continuous variables with the response categories of the questions in the questionnaire. Click here for file

Additional file 9 **Table S8. **Significant binary variables of the questionnaire in TE male patients. Click here for file

Additional file 10 **Table S9. **Significant binary variables of the questionnaire in SE male patients. Click here for file

Additional file 11 **Table S10. **Significant binary variables of the questionnaire in SY male patients. Click here for file

Additional file 12 **Table S11. **Significant binary variables of the questionnaire in TE female patients. Click here for file

Additional file 13 **Table S12. **Significant binary variables of the questionnaire in SE female patients. Click here for file

Additional file 14 **Table S13. **Significant binary variables of the questionnaire in SY female patients. Click here for file

Additional file 15 **Table S14. **Selected variables and estimated parameters for face (male). Click here for file

Additional file 16 **Table S15. **Selected variables and estimated parameters for face (female). Click here for file

Additional file 17 **Table S16. **Selected variables and estimated parameters for body shape (male). Click here for file

Additional file 18 **Table S17. **Selected variables and estimated parameters for body shape (female). Click here for file

Additional file 19 **Table S18. **Selected variables and estimated parameters for voice (male). Click here for file

Additional file 20 **Table S19. **Selected variables and estimated parameters for voice (female). Click here for file

Additional file 21 **Table S20. **Selected variables and estimated parameters for questionnaire (male). Click here for file

Additional file 22 **Table S21. **Selected variables and estimated parameters for questionnaire (female). Click here for file
