# Supplementary material for: Development of an integrated Sasang constitution diagnosis method using face, body shape, voice, and questionnaire information
Source: BMC Complement Altern Med. 2012 Jul 4;12:85. doi: 10.1186/1472-6882-12-85 (PMC3502327; doi:10.1186/1472-6882-12-85)
Supplement: Additional file 1 — Table S1. Population characteristics of the participants. [file 1472-6882-12-85-S1.docx]

Table S1. Population characteristics of the participants

|  | | AGE  (yr) | Height  (cm) | Weight  (kg) | Systolic blood pressure (mm Hg) | Diastolic blood pressure (mm Hg) | BMI (kg/m^2^) | Occupation (%) | | | Education (%) | Married (%) |
| --- | --- | --- | --- | --- | --- | --- | --- | --- | --- | --- | --- | --- |
|  |  |  |  |  |  |  |  | white collar | blue collar | house & etc. | > 9 yrs | yes |
| Male (1,077) | TE (473) | 47.8 ±16.9† | 169.6 ±6.9 | 73.9 ±11.2 | 125.8 ±14.2 | 80.1 ±10.5 | 25.6 ±3.0 | 226 (49.1) | 94 (20.4) | 140 (30.4) | 358 (77.3) | 367 (78.7) |
|  | SE (245) | 42.0 ±16.3 | 168.9 ±7.9 | 61.6 ±9.4 | 118.3 ±15.3 | 76.2 ±11.6 | 21.5 ±2.6 | 126 (52.5) | 37 (15.4) | 77 (32.0) | 189 (77.7) | 164 (67.4) |
|  | SY (339) | 49.1 ±15.5 | 168.8 ±6.3 | 67.0 ±9.3 | 120.9 ±14.3 | 78.9 ±10.4 | 23.5 ±2.7 | 152 (46.3) | 86 (26.2) | 90 (27.4) | 242 (72.4) | 271 (80.8) |
|  | TY (20) | 39.5 ±16.8 | 171.8 ±5.2 | 62.8 ±9.4 | 116.6 ±14.6 | 74.2 ±9.6 | 21.24 ±2.8 | 13 (65.0) | 2 (10.0) | 5 (25.0) | 18 (90.0) | 13 (65.0) |
| Female (1,896) | TE (701) | 49.4 ±16.0 | 157.4 ±6.0 | 62.3 ±9.2 | 121.0 ±16.4 | 77.6 ±11.3 | 25.1 ±3.3 | 182 (26.8) | 67 (9.80) | 429 (63.2) | 396 (57.7) | 562 (81.4) |
|  | SE (501) | 45.5 ±15.6 | 157.9 ±5.9 | 52.4 ±6.6 | 115.3 ±15.4 | 74.1 ±10.8 | 20.1 ±2.6 | 149 (29.9) | 36 (7.20) | 312 (62.7) | 357 (71.6) | 380 (76.3) |
|  | SY (649) | 45.9 ±14.8 | 156.8 ±6.1 | 55.1 ±7.2 | 115.8 ±14.1 | 74.49 ±10.4 | 22.4 ±2.7 | 227 (35.6) | 65 (10.2) | 345 (54.1) | 435 (67.4) | 517 (80.0) |
|  | TY (45) | 43.4 ±12.0 | 158.9 ±5.6 | 53.3 ±7.3 | 113.1 ±12.8 | 74.4 ±9.3 | 21.1 ±2.9 | 16 (36.3) | 3 (6.80) | 25 (56.8) | 35 (77.7) | 39 (86.6) |

†  $\bar{x}+SD$ for all such values, SD: standard deviation
