# Supplementary figures and images for: Development of an integrated Sasang constitution diagnosis method using face, body shape, voice, and questionnaire information
Source: BMC Complement Altern Med. 2012 Jul 4;12:85. doi: 10.1186/1472-6882-12-85 (PMC3502327; doi:10.1186/1472-6882-12-85)

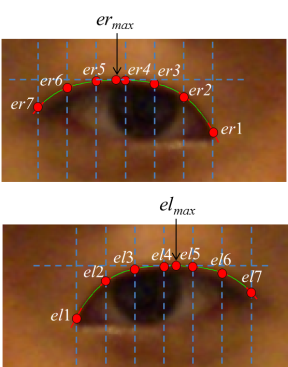

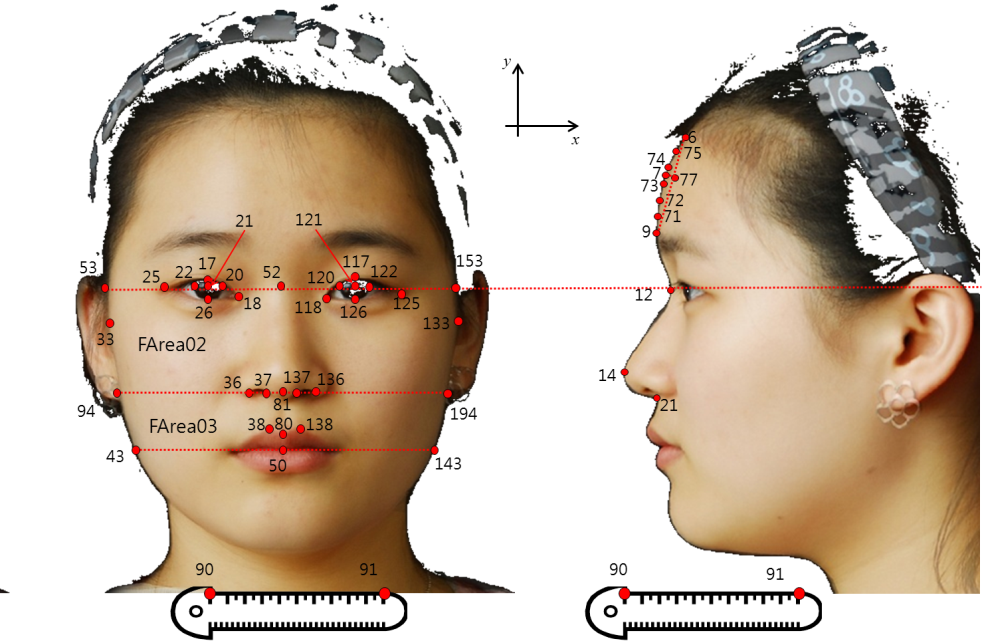


Figure S1. Facial points used to calculated candidate feature variables

Supplement: Additional file 2 — Figure S1. Facial points used to calculated candidate feature variables. [file 1472-6882-12-85-S2.docx]
