# Supplementary material for: Development of an integrated Sasang constitution diagnosis method using face, body shape, voice, and questionnaire information
Source: BMC Complement Altern Med. 2012 Jul 4;12:85. doi: 10.1186/1472-6882-12-85 (PMC3502327; doi:10.1186/1472-6882-12-85)
Supplement: Additional file 3 — Table S2. Feature variables expressing the facial characteristics. [file 1472-6882-12-85-S3.docx]

Table S2. Feature variables expressing the facial characteristics

|  | Size-related variables | Shape-related variables |
| --- | --- | --- |
| Face shape | ∙ Width: FDH(33,133), FD(43,143),  FD(53,153), FD(94,194)  ∙ Height: FDV(81,50), FDV(52,50)  ∙ Area: FArea02, FArea03 | ∙Ratio: FDH(33,133)/FD(43,143)  FDH(33,133)/FDV(52,50)  FD(43,143)/FDV(52,50)  ∙Angle: FA(18,25,43) |
| Forehead | ∙ Height: PDV(6,9), PDV(7,9),  PDV(6,7), PDV(9,12) | ∙ Angle: PA(7,6), PA(72,73), PA(9,12)  ∙ Ratio: PDV(7,9)/PDV(6,9)  ∙ Depth: PDH(6,7), PDH(9,12)  ∙ Distance: PD(7,77) |
| Eye | ∙ Width: FDH(18,118), FDH(25,125)  ∙ Height: FD(17,26), FD(117,126)  ∙ Distance: FD(18,25) , FD(118,125) | ∙ Angle: FA(25,17), FA(125,117),  FA(18,17), FA(118,117)  ∙ Distance: FD(17,25), FD(117,125)  ∙ Ratio: FD(17,26)/FD(18,25), FD(117,126)/FD(118,125),  (FD(18,25)+FD(118,125))/FDH(33,133) |
| Upper eyelid | ∙ Width: FDH(*el*1, *el_max_*),  FDH(*er*1, *er_max_*) | ∙ Angle: FST(*el*1) ~ FST(*el*7), FST(*er*1) ~ FST(*er*7)  ∙ Ratio: FDH(*el*1, *el_max_*)/FDH(*el*1, *el*7),  FDH(*er*1, *er_max_*)/FDH(*er*1, *er*7),  FDV(*el*7, *el*1)/FDH(*el*7, *el*1),  FDV(*er*7, *er*1)/FDH(*er*7, *er*1),  FDV(*el_max_*,*el*1)/FDH(*el_max_*, *el*1)  FDV(*er_max_*,*er*1)/FDH(*er_max_*, *er*1)  ∙ Curvature: FCLE*_avg_*, FCLE*_max_*, FCRE*_avg_*, FCRE*_max_* |
| Nose | ∙ Width: FDH(36,136)  ∙ Height: FDV(52,81), PD(12,21)  PDV(12,14), PDV(14,21)  ∙ Depth: PDH(12,14), PDH(14,21)  ∙ Area: PAR(12,14,21) | ∙ Angle: PA(14,12), PA(14,21), PA(12,14,21) |
| Mouth | ∙ Height: FDV(38,50), FDV(138,50) |  |

*FD(*n_1_,n_2_*) [or PD(*n_1_,n_2_*)]: distance between point *n_1_* and *n_2_* in a frontal (or profile) image

*FDH(*n_1_,* *n*_2_) [or PDH(*n_1_,* *n*_2_)]: horizontal distance between *n_1_* and *n_2_* in a frontal (or profile) image

*FDV(*n_1_,* *n*_2_) [or PDV(*n_1_,* *n*_2_)]: vertical distance between *n_1_* and *n_2_* in a frontal (or profile) image

*FA(*n_1_,n_2_*) [or PA(*n_1_,n_2_*)]: angle between the line through two points *n_1_* and *n_2_* and a horizontal line in a frontal (or profile) image

*FA(*n_1_,n_2_,n_3_* ) [or PA(*n_1_,n_2_,n_3_* )]: angle between three points, *n_1_*, *n_2_*, and *n_3_*, in a frontal(or profile) image

*PAR(*n_1_,n_2_,n_3_* ): area of the triangle formed by three points, *n_1_*, *n_2_*, and *n_3_*, in a profile image

*FST(*n_1_*): slope of the tangent line at a point *n_1_* in a frontal image

*FCLE*_avg_* [or FCRE*_avg_*]: average curvature of the left (or right) upper eyelid contour in a frontal image

*FCLE*_max_* [or FCRE*_max_*]: maximum curvature of the left (or right) upper eyelid contour in a frontal image
