# Supplementary material for: Development of an integrated Sasang constitution diagnosis method using face, body shape, voice, and questionnaire information
Source: BMC Complement Altern Med. 2012 Jul 4;12:85. doi: 10.1186/1472-6882-12-85 (PMC3502327; doi:10.1186/1472-6882-12-85)
Supplement: Additional file 4 — Table S3. Measurement methods of eight circumferences for body shape. [file 1472-6882-12-85-S4.docx]

Table S3. Measurement methods of eight circumferences for body shape

| Eight circumference parts | Measuring method |
| --- | --- |
| Forehead circumference  (FC) | A subject sits on a chair with his/her back straightened. An observer holds the ‘0 point’ with one hand. He/she wraps the tapeline around subject’s forehead, passing the glabella and the opisthion, and overlaps the tapeline and measures. The observer should apply sufficient pressure on the subject’s hair. |
| Neck circumference  (NC) | A subject sits on a chair with his/her back straightened. He/she maintains his/her forehead position parallel to the floor. An observer stands in front of the subject and wraps the measuring tape around the neck, passing the area between the thyroid cartilage and the cricoid cartilage. The shortest distance is recorded. |
| Axillary circumference  (AC) | A subject undresses his/her upper body and stands up straight with balanced pressure on the right and left feet. An observer stands in front of the subject. The subject raises his/her arms laterally, and the observer wraps measuring tape around the subject’s upper body to pass the right and left axilla and the midpoint of the chuhndohl (天突) and the joongjuhng (中庭). The subject lowers his/her arms in a natural way, and the circumference is recorded. |
| Chest circumference  (CC) | A subject undresses his/her upper body and stands up straight with balanced pressure on the right and left feet. An observer stands in front of the subject. The subject raises his/her arms laterally, and the observer wraps measuring tape around the subject’s upper body to pass the right and left nipple points. The subject lowers his/her arms in a natural way, and the circumference is recorded. |
| Rib  circumference  (RC) | A subject undresses his/her upper body and stands up straight with balanced pressure on the right and left feet. An observer stands in front of the subject. The subject raises his/her arms laterally, and the observer wraps measuring tape around the subject’s upper body to pass the right and left seventh and eighth prominence of the costochondral junction. The subject lowers his/her arms in a natural way, and the circumference is recorded. |
| Waist circumference  (WC) | A subject undresses his/her upper body and stands up straight with balanced pressure on the right and left feet. An observer stands in front of the subject. The subject raises his/her arms laterally, and the observer wraps measuring tape around the subject’s upper body to cover the umbilicus. The subject lowers his/her arms in a natural way, and the circumference is recorded |
| Pelvic circumference  (PC) | A subject undresses his/her upper body and sufficiently rolls down the pants and underwear to expose measuring areas. He/she stands up straight with balanced pressure on the right and left feet. The subject crosses his/her arms across the chest. An observer stands in front of the subject and wraps the measuring tape to pass the right and left ASIS. The circumference is recorded. |
| Hip circumference  (HC) | A subject undresses his/her upper body and sufficiently rolls down the pants and underwear to expose measuring areas. He/she stands up straight with balanced pressure on the right and left feet. The subject crosses his/her arms across the chest. An observer stands in front of the subject and wraps the measuring tape to pass right over the pubis. The circumference is recorded. |
