# Supplementary material for: Development of an integrated Sasang constitution diagnosis method using face, body shape, voice, and questionnaire information
Source: BMC Complement Altern Med. 2012 Jul 4;12:85. doi: 10.1186/1472-6882-12-85 (PMC3502327; doi:10.1186/1472-6882-12-85)
Supplement: Additional file 5 — Table S4. Description of vowel features. [file 1472-6882-12-85-S5.docx]

Table S4. Description of vowel features

| Vowel features | Description |
| --- | --- |
|  |  |
| *x*F0, *x*STD | Average pitch frequency and standard deviation of pitch |
| *x*T0, *x*JITA, *x*JITT | Average period, absolute jitter and percentage of jitter |
| *x*PPQ, *x*RAP | Pitch perturbation quotient and relative average perturbation |
| *x*SHDB, *x*SHIM | Shimmer in dB and percentage of Shimmer |
| *x*APQ | Amplitude perturbation quotient |
| *x*DTF0 | Average difference of F0 over the time interval |
| *x*ENG, *x*PW | Energy and power |
| *x*F1, *x*F2, *x*F3, *x*F4 | Formant frequencies |
| *x*BW1, *x*BW2 | Bandwidths of 1st and 2nd formant frequencies |
| *x*F2/*x*F1, *x*F3/*x*F1, *x*F4/*x*F1, *x*F3/*x*F2, *x*F4/*x*F2, *x*F4/*x*F3 | Ratios of formant frequencies |
| *x*MFCC1 ~ 13 | 13 Mel-Frequency Cepstral Coefficients |
| *x*LPR1 | Log of power ratio of frequency range 60-240 Hz to 240-960 Hz |
| *x*LPR2 | Log of power ratio of frequency range 240-960 Hz to 960-3840 Hz |
| *x*LPR3 | Log of power ratio of frequency range 60-240 Hz to 960-3840 Hz |

*x*$\in${a, e, i, o, u}
