# Supplementary material for: Development of an integrated Sasang constitution diagnosis method using face, body shape, voice, and questionnaire information
Source: BMC Complement Altern Med. 2012 Jul 4;12:85. doi: 10.1186/1472-6882-12-85 (PMC3502327; doi:10.1186/1472-6882-12-85)
Supplement: Additional file 6 — Table S5. Description of sentence features. [file 1472-6882-12-85-S6.docx]

Table S5. Description of sentence features

| Sentence features | Description |
| --- | --- |
|  |  |
| sF0, sFSTD | Average pitch frequency and standard deviation of pitch |
| sF10, sF50, sF90 | 10^th^, 50^th^, and 90^th^ percentiles of pitch distribution |
| sFHL | Ratio of (sF90-sF50) to (sF50-sF10) |
| sI0, sISTD | Average intensity and standard deviation of intensity |
| sI10, sI50, sI90 | 10^th^, 50^th^, and 90^th^ percentiles of intensity distribution |
| sIHL | Ratio of (sI90-sI50) to (sI50-sI10) |
| sCORR | Pearson correlation coefficient between frequency and intensity |
| sSPD | Elapsed time of reading |
| sLPR1 | Log of power ratio of frequency range 60-240 Hz to 240-960 Hz |
| sLPR2 | Log of power ratio of frequency range 240-960 Hz to 960-3840 Hz |
| sLPR3 | Log of power ratio of frequency range 60-240 Hz to 960-3840 Hz |
