# Supplementary material for: Development of an integrated Sasang constitution diagnosis method using face, body shape, voice, and questionnaire information
Source: BMC Complement Altern Med. 2012 Jul 4;12:85. doi: 10.1186/1472-6882-12-85 (PMC3502327; doi:10.1186/1472-6882-12-85)
Supplement: Additional file 7 — Table S6. List of questions. [file 1472-6882-12-85-S7.docx]

Table S6. List of questions

| **Personality Questionnaire Ⅰ**  **Select (**✔**) among ①②③ for the following questions. (② lies between the two extremes.)** | | | | | | | | | | | | | | | | | |
| --- | --- | --- | --- | --- | --- | --- | --- | --- | --- | --- | --- | --- | --- | --- | --- | --- | --- |
| ***[Example] Do you give up easily or persevere?*** | | | | | | | | ***Give up*** | | | ***①✔*** | | ***②*** | | ***③*** | | ***Persevere*** |
| 1. Are you bold or delicate? | | | | | | | | Bold | | | ① | | ② | | ③ | | Delicate |
| 2. Are your actions quick or slow? | | | | | | | | Quick | | | ① | | ② | | ③ | | Slow |
| 3. Are you active or passive? | | | | | | | | Active | | | ① | | ② | | ③ | | Passive |
| 4. Are you direct or indirect in communication? | | | | | | | | Direct | | | ① | | ② | | ③ | | Indirect |
| 5. Do you give up easily or persevere? | | | | | | | | Give up | | | ① | | ② | | ③ | | Persevere |
| 6. Are you an extrovert or introvert? | | | | | | | | Extrovert | | | ① | | ② | | ③ | | Introvert |
| 7. Are you energetic or quiet? | | | | | | | | Energetic | | | ① | | ② | | ③ | | Quiet |
| 8. Do you find it easy or difficult to make decisions? | | | | | | | | Easy | | | ① | | ② | | ③ | | Difficult |
| 9. Are you masculine or feminine? | | | | | | | | Masculine | | | ① | | ② | | ③ | | Femiine |
| 10. Are you impatient or patient? | | | | | | | | Impatient | | | ① | | ② | | ③ | | Patient |
| 11. Are you emotionally volatile or calm? | | | | | | | | Volatile | | | ① | | ② | | ③ | | Calm |
| 12. Are you an open or private person? | | | | | | | | Open | | | ① | | ② | | ③ | | Private |
| 13. Do you express or hide your opinions? | | | | | | | | Express | | | ① | | ② | | ③ | | Hide |
| 14. Do you get excited or remain calm? | | | | | | | | Excited | | | ① | | ② | | ③ | | Calm |
| 15. Are you careless or careful? | | | | | | | | Careless | | | ① | | ② | | ③ | | Careful |
| **Habits Questionnaire** | | | | | | | | | | | | | | | | | |
| Please answer the following on habits in the **past 6 months** based on your **usual lifestyle**. Ask the person-in-charge if you are unsure. You can **check more than once for questions marked by (*)**. | | | | | | | | | | | | | | | | | |
| **Meal** | Do you have regular meals? _1_☐Yes _2_☐No | | | | | | | | | | | | | | | | |
|  | How many meals do you usually eat a day? ( )times/day (e.g.) 2 times/day | | | | | | | | | | | | | | | | |
|  | How much do you eat? _1_☐A lot _2_☐Moderate _3_☐A little _4_☐Irregular | | | | | | | | | | | | | | | | |
|  | How fast do you eat? _1_☐Fast _2_☐Moderate _3_☐Slow | | | | | | | | | | | | | | | | |
|  | How much time do you spend for each meal? Approx. ( )minutes/meal (e.g.) 7 minutes, 15 minutes, etc. | | | | | | | | | | | | | | | | |
| **Digestion** | Do you digest well? _1_☐Yes  _2_☐No **⇨** Do you experience discomfort from indigestion? _1_☐Yes _2_☐No | | | | | | | | | | | | | | | | |
|  | How is your appetite? _1_☐Very good _2_☐Good _3_☐Fair _4_☐Not good | | | | | | | | | | | | | | | | |
|  | Below are questions related to digestion. Do you experience the following symptoms? Check all that apply.   \| **Item** \| **Often** \| **Sometimes** \| **Not at all** \| \| --- \| --- \| --- \| --- \| \| Upset stomach \| _1_☐ \| _2_☐ \| _3_☐ \| \| Vomit \| _1_☐ \| _2_☐ \| _3_☐ \| \| Queasiness \| _1_☐ \| _2_☐ \| _3_☐ \| \| Famished \| _1_☐ \| _2_☐ \| _3_☐ \| \| Burp \| _1_☐ \| _2_☐ \| _3_☐ \| \| Acid reflux \| _1_☐ \| _2_☐ \| _3_☐ \| \| Indigestion \| _1_☐ \| _2_☐ \| _3_☐ \| \| Nausea \| _1_☐ \| _2_☐ \| _3_☐ \| \| Stomachache (digestion) \| _1_☐ \| _2_☐ \| _3_☐ \| \| Bloated stomach \| _1_☐ \| _2_☐ \| _3_☐ \| | | | | | | | | | | | | | | | | |
| **Perspiration** | How much do you perspire? _1_☐A lot _2_☐Moderate _3_☐A little _4_☐None | | | | | | | | | | | | | | | | |
|  | Below are questions related to perspiration. Specify the amount of perspiration according to the given situation.   \| **Item** \| **A lot** \| **Moderate** \| **A little** \| **None** \| \| --- \| --- \| --- \| --- \| --- \| \| Hot weather \| _1_☐ \| _2_☐ \| _3_☐ \| _4_☐ \| \| Exercise \| _1_☐ \| _2_☐ \| _3_☐ \| _4_☐ \| \| Nervousness \| _1_☐ \| _2_☐ \| _3_☐ \| _4_☐ \| \| Daily life \| _1_☐ \| _2_☐ \| _3_☐ \| _4_☐ \| | | | | | | | | | | | | | | | | |
|  | How do you feel after respiration?  _1_☐Refreshed _2_☐Tired _3_☐No feeling | | | | | | | | | | | | | | | | |
|  | (*)Where do you usually perspire? (Check all that apply)  _1_☐Head and face _2_☐Neck _3_☐Chest and armpits _4_☐Back _5_☐Hands _6_☐Feet _7_☐Groin _8_☐All over | | | | | | | | | | | | | | | | |
|  | (*)Have you experienced abnormal perspiration? (Check all that apply)  _1_☐No _2_☐Night sweats _3_☐Day sweats _4_☐Sweat during meals | | | | | | | | | | | | | | | | |
| **Excrement** | Do you excrete regularly? _1_☐Yes _2_☐No | | | | | | | | | | | | | | | | |
|  | How often do you excrete? ( )times/( )day (e.g.) 1 time/day, 2 times/3 days, etc. | | | | | | | | | | | | | | | | |
|  | How fast do you excrete? _1_☐Fast _2_☐Normal _3_☐Slow | | | | | | | | | | | | | | | | |
|  | How much time do you spend for each excrement? ( )minutes/session (e.g.) 5 minutes, 7 minutes, 10 minutes, etc. | | | | | | | | | | | | | | | | |
|  | (*)What color is your excrement? (Check all that apply)  _1_☐Yellow (Gold) _2_☐Brown _3_☐Black _4_☐Gray _5_☐Maroon (Blood) | | | | | | | | | | | | | | | | |
|  | How thick is your excrement? _1_☐Thin _2_☐Normal _3_☐Thick | | | | | | | | | | | | | | | | |
|  | How hard is your excrement? _1_☐Hard _2_☐Normal _3_☐Soft | | | | | | | | | | | | | | | | |
|  | How is your excrement condition?  _1_☐Diarrhea _2_☐Constipation _3_☐Diarrhea/Constipation _4_☐Depends on food consumption _5_☐Normal | | | | | | | | | | | | | | | | |
|  | What does your excrement look like? Select an image that is closest in appearance. | | | | | | | | | | | | | | | | |
|  | _1_☐ _2_☐ _3_☐ _4_☐ _5_☐ _6_☐ _7_☐ | | | | | | | | | | | | | | | | |
|  | 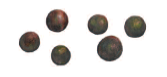 | | 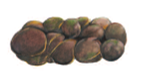 | | | | 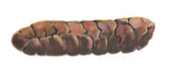 | | 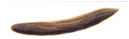 | | | 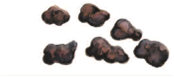 | | 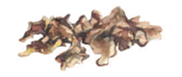 | | 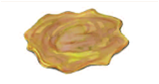 | |
|  | Firm, round pellets | | Sausage-shaped with a hard and uneven surface | | | | Sausage-shaped with cracks | | Soft chocolate bar | | | Mushy pasta | | Bits of gruel | | Watery diarrhea | |
|  | Do you feel refreshed during or after excrement?  _1_☐Yes _2_☐No **⇨** Is this accompanied by any pain? _1_☐Yes _2_☐No | | | | | | | | | | | | | | | | |
|  | Below are questions related to excrement. Do you experience the following symptoms? Check all that apply.   \| **Item** \| **Often** \| **Sometimes** \| **Not at all** \| \| --- \| --- \| --- \| --- \| \| Watery excrement \| _1_☐ \| _2_☐ \| _3_☐ \| \| Stiff excrement \| _1_☐ \| _2_☐ \| _3_☐ \| \| Unable to excrete on some days \| _1_☐ \| _2_☐ \| _3_☐ \| \| Lower abdominal pain \| _1_☐ \| _2_☐ \| _3_☐ \| \| Abdominal expansion (filled with gas) \| _1_☐ \| _2_☐ \| _3_☐ \| \| Abdominal discomfort \| _1_☐ \| _2_☐ \| _3_☐ \| \| Tension during excretion \| _1_☐ \| _2_☐ \| _3_☐ \| \| Feeling of residual excretion \| _1_☐ \| _2_☐ \| _3_☐ \| | | | | | | | | | | | | | | | | |
| **Urine** | | How often do you urinate during the day ( )times/day (e.g.) 2 times, 5 times, 10 times, etc. | | | | | | | | | | | | | | | |
|  |  | How often do you get up at night to urinate? ( )times/day (e.g.) 0 times, 1 time, 2 times, etc. | | | | | | | | | | | | | | | |
|  |  | How strong is your urine stream? _1_☐Strong _2_☐Normal _3_☐Weak | | | | | | | | | | | | | | | |
|  |  | (*)How is the color and turbidity of your urine? (Check all that apply)  _1_☐Turbid _2_☐Clear _3_☐Yellow _4_☐Reddish _5_☐Foamy | | | | | | | | | | | | | | | |
|  |  | Below are questions related to urine. Do you experience the following symptoms? Check all that apply.   \| **Item** \| **Often** \| **Sometimes** \| **Not at all** \| \| --- \| --- \| --- \| --- \| \| Urinary pain (including discomfort) \| _1_☐ \| _2_☐ \| _3_☐ \| \| Frequent urination \| _1_☐ \| _2_☐ \| _3_☐ \| \| Difficult to begin urination \| _1_☐ \| _2_☐ \| _3_☐ \| \| Feeling of residual urine \| _1_☐ \| _2_☐ \| _3_☐ \| \| Urinary incontinence \| _1_☐ \| _2_☐ \| _3_☐ \| | | | | | | | | | | | | | | | |
| **Cold and Heat** | | Which do you dislike more, cold or heat? _1_☐Cold _2_☐Heat _3_☐Both _4_☐Neither | | | | | | | | | | | | | | | |
|  |  | Are your hands cold or warm? | | | | | | | | _1_☐Warm _2_☐Normal _3_☐Cold _4_☐Not sure | | | | | | | |
|  |  | Are your feet cold or warm? | | | | | | | | _1_☐Warm _2_☐Normal _3_☐Cold _4_☐Not sure | | | | | | | |
|  |  | Is your stomach cold or warm? | | | | | | | | _1_☐Warm _2_☐Normal _3_☐Cold _4_☐Not sure | | | | | | | |
|  |  | Below are questions related to head and cold. Check all that apply.   \| **Item** \| **Often** \| **Sometimes** \| **Not at all** \| \| --- \| --- \| --- \| --- \| \| I like warm temperatures \| _1_☐ \| _2_☐ \| _3_☐ \| \| My stomach is cold and chilly \| _1_☐ \| _2_☐ \| _3_☐ \| \| My hands and feet become cold easily \| _1_☐ \| _2_☐ \| _3_☐ \| \| My face looks pale \| _1_☐ \| _2_☐ \| _3_☐ \| \| My urine is clear and transparent \| _1_☐ \| _2_☐ \| _3_☐ \| \| I dislike drinking water even when I feel thirsty \| _1_☐ \| _2_☐ \| _3_☐ \| \| I like cool temperatures \| _1_☐ \| _2_☐ \| _3_☐ \| \| My body is feverish and hot \| _1_☐ \| _2_☐ \| _3_☐ \| \| My hands and feet are hot and stuffy \| _1_☐ \| _2_☐ \| _3_☐ \| \| I get bloodshot eyes and a red face \| _1_☐ \| _2_☐ \| _3_☐ \| \| I feel thirsty and crave cold water \| _1_☐ \| _2_☐ \| _3_☐ \| \| My urine is dark \| _1_☐ \| _2_☐ \| _3_☐ \| | | | | | | | | | | | | | | | |
| **Water Consumption** | | How much water do you drink? _1_☐A lot _2_☐Moderate _3_☐A little _4_☐A little but often | | | | | | | | | | | | | | | |
|  |  | How much water do you drink a day in 200 ml glasses? ( )glasses/day | | | | | | | | | | | | | | | |
|  |  | How is the temperature of your drinking water?  _1_☐Usually warm water _2_☐Usually cold water _3_☐I don't care | | | | | | | | | | | | | | | |
| **Sleep** | | How much do you dream? _1_☐Most of the time _2_☐A lot of the time _3_☐A little of the time _4_☐None of the time | | | | | | | | | | | | | | | |
|  |  | (*)How are your sleeping habits? (Check all that apply)  _1_☐Sleep soundly _2_☐Trouble falling asleep _3_☐Easily interrupted _4_☐Frequent napping | | | | | | | | | | | | | | | |
|  |  | How long is your actual sleeping time?   \|  \|  \| \| --- \| --- \|   hours   \|  \|  \| \| --- \| --- \|   minutes  (Do not include the time taken to fall asleep) | | | | | | | | | | | | | | | |
|  |  | How would you evaluate the quality of your sleep? _1_□Very good _2_□Good _3_□Bad _4_☐Very bad | | | | | | | | | | | | | | | |
| **Symptoms Questionnaire** | | | | | | | | | | | | | | | | | |
| Please answer the following for **symptoms** that you have experienced in the **past 6 months**. Ask the person-in-charge if you are unsure. You can **check more than once for questions marked by (*)**. | | | | | | | | | | | | | | | | | |
| **Head** | | (*)Do you experience any discomfort in the head?  _1_☐Forehead _2_☐Side _3_☐Back _4_☐All over _5_☐No (Skip to the next section) | | | | | | | | | | | | | | | |
|  |  | (*)If you do experience discomfort, what are the symptoms?  _1_☐Heaviness _2_☐Fever _3_☐Prickling _4_☐Nausea _5_☐Emptiness | | | | | | | | | | | | | | | |
| **Neck** | | (*)Do you experience any discomfort below the head?  _1_☐Shoulders or back of the neck _2_☐Back _3_☐Waist _4_☐Knees _5_☐No | | | | | | | | | | | | | | | |
| **Eyes** | | (*)Do you experience any discomfort in the eyes?  _1_☐Pain _2_☐Dryness _3_☐Bloodshot _4_☐Fatigue _5_☐Strain _6_☐No | | | | | | | | | | | | | | | |
| **Mouth/Neck** | | (*)Do you experience any discomfort in the mouth?  _1_☐Dry mouth _2_☐Bad breath _3_☐Frequent blisters _4_☐Cough _5_☐Phlegm _6_☐Sore throat _7_☐No | | | | | | | | | | | | | | | |
| **Chest** | | (*)Do you experience any discomfort in the chest?  _1_☐Stuffiness _2_☐Pain _3_☐Pounding _4_☐Palpitation _5_☐Frequent sighing _6_☐No | | | | | | | | | | | | | | | |
| **Skin** | | (*)Do you experience any discomfort in the skin? _1_☐Dryness _2_☐Itch _3_☐No | | | | | | | | | | | | | | | |
| **Fatigue** | | How much fatigue do you have?  _1_☐None (Skip to the next section) | | | | | | | | | | | | | | | |
|  |  | _2_☐A little  _3_☐Moderate  _4_☐Serious | | } | **⇨** | (*)If yes, when do you feel the most fatigue? (Check all that apply)  _1_☐When getting up _2_☐Morning _3_☐Afternoon _4_☐Night _5_☐All day | | | | | | | | | | | |
| **Cold** | | (*)What symptoms do you have during a cold?  _1_☐None _2_☐Fever _3_☐Headache _4_☐Dizziness _5_☐Runny or stuffy nose _6_☐Deafened _7_☐Cough (phlegm) _8_☐Neck pain _9_☐Body ache _10_☐Numb arm and legs _11_☐Loss of appetite or indigestion | | | | | | | | | | | | | | | |
| **Condition** | | (*)Where do you experience discomfort when you are unwell? _1_☐Perspiration _2_☐Digestion _3_☐Excrement _4_☐Urine _5_☐Others( ) _6_☐None | | | | | | | | | | | | | | | |
| **Others** | | (*)Specify any other symptoms that you experience.  _1_☐Swelling _2_☐Forgetfulness _3_☐Dizziness _4_☐Weakness in lower legs _5_☐Feverish with swollen joints  _6_☐Others( ) _7_☐None | | | | | | | | | | | | | | | |
