# Supplementary material for: Development of an integrated Sasang constitution diagnosis method using face, body shape, voice, and questionnaire information
Source: BMC Complement Altern Med. 2012 Jul 4;12:85. doi: 10.1186/1472-6882-12-85 (PMC3502327; doi:10.1186/1472-6882-12-85)
Supplement: Additional file 8 — Table S7. The procedure of generating continuous variables with the response categories of the questions in the questionnaire. [file 1472-6882-12-85-S8.docx]

Table S7. The procedure of generating continuous variables with the response categories
of the questions in the questionnaire [31]

| 1. Subgrouping:   Within each sex, the training set was repeatedly divided into two subgroups in three different ways based on SC type: “an SC type” and “the remaining two,” i.e., TE vs. non-TE, SE vs. non-SE, and SY vs. non-SY.   1. Searching significant binary variables:   Categories in each variable were repeatedly re-categorized into two categories, “a category selected” and “remaining others,” leaving us with binary variables that were as numerous as the number of categories in the question. As an example, if a question had five response categories, there would be five binary variables created. A chi-square test was applied to find any significant binary variables between each pair of the two subgroups with a p value < 0.001.   1. Weight assignment:   An idea of assigning weights using the logarithm of the p value was employed such that a more significant binary variable corresponded to that variable having a larger weight. Either positive or negative weights were assigned to each category of variable, depending on whether the association in the corresponding two by two table was positive or negative.   1. Calculating feature variables:   The continuous feature variables *Q_TE_*, *Q_SE_*, and *Q_SY_* were calculated by totaling the weights over all categories so as to show the significance for each constitutional subgroup.  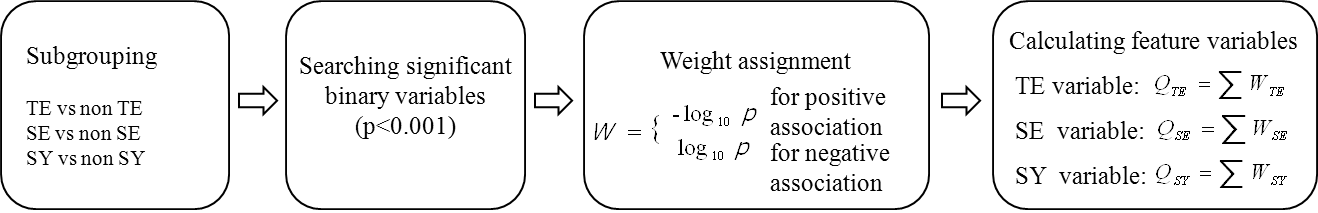 |
| --- |
