# Supplementary material for: Development of an integrated Sasang constitution diagnosis method using face, body shape, voice, and questionnaire information
Source: BMC Complement Altern Med. 2012 Jul 4;12:85. doi: 10.1186/1472-6882-12-85 (PMC3502327; doi:10.1186/1472-6882-12-85)
Supplement: Additional file 9 — Table S8. Significant binary variables of the questionnaire in TE male patients. [file 1472-6882-12-85-S9.docx]

Table S8. Significant binary variables of the questionnaire in TE male patients

| Question | | Binary variable  (Answer) | Weight | N |
| --- | --- | --- | --- | --- |
| Personality | Action | Quick | -6.467 | 145 |
| Meal | Meal Size | Small | -3.274 | 36 |
|  | Eating Speed | Fast | 3.622 | 229 |
| Digestion | Appetite Sensation | Good | 4.952 | 219 |
|  | Appetite Sensation | Moderate | -4.975 | 81 |
| Perspiration | Amount | A lot | 4.861 | 150 |
|  | Feeling after Perspiration | Tired | -4.997 | 56 |
| Cold and Heat | Dislike | Hot | -6.129 | 107 |
|  | Dislike | Cold | 9.239 | 173 |
|  | Hand | Warm | 5.27 | 191 |
|  | Hand | Cold | -5.202 | 55 |
|  | Foot | Warm | 4.496 | 140 |
|  | Foot | Cold | -4.724 | 77 |
| In Bad | Digestion Problem | No | 4.427 | 270 |
| Condition | Digestion Problem | Yes | -4.427 | 76 |
