# Supplementary material for: Development of an integrated Sasang constitution diagnosis method using face, body shape, voice, and questionnaire information
Source: BMC Complement Altern Med. 2012 Jul 4;12:85. doi: 10.1186/1472-6882-12-85 (PMC3502327; doi:10.1186/1472-6882-12-85)
Supplement: Additional file 10 — Table S9. Significant binary variables of the questionnaire in SE male patients. [file 1472-6882-12-85-S10.docx]

Table S9. Significant binary variables of the questionnaire in SE male patients

|  | Question | Binary variable  (Answer) | Weight | N |
| --- | --- | --- | --- | --- |
| Personality | Bold or Delicate | Bold | -5.496 | 12 |
|  | Bold or Delicate | Delicate | 3.507 | 101 |
|  | Active or Passive | Active | -5.655 | 66 |
|  | Active or Passive | Passive | 3.04 | 39 |
|  | Extrovert or Introvert | Extrovert | -3.274 | 25 |
|  | Energetic or Quiet | Energetic | -5.632 | 51 |
|  | Energetic or Quiet | Quiet | 6.129 | 91 |
|  | Easy or Difficult to Make a Decision | Easy | -3.53 | 46 |
|  | Masculine or Feminine | Masculine | -6.669 | 63 |
|  | Masculine or Feminine | Moderate | 3.157 | 88 |
| Digestion | Good Digestion | Yes | -4.952 | 133 |
|  | Good Digestion | No | 4.952 | 56 |
|  | Discomfort | Null | -4.884 | 132 |
|  | Discomfort | No | 3.483 | 21 |
|  | Appetite Sensation | Good | -7.051 | 71 |
|  | Appetite Sensation | Moderate | 6.309 | 88 |
| Perspiration | Amount | A lot | -4.587 | 42 |
|  | Amount | A little | 3.227 | 66 |
|  | Feeling after Perspiration | Refreshed | -3.669 | 60 |
|  | Feeling after Perspiration | Tired | 6.039 | 70 |
| Cold and Heat | Dislike | Hot | 6.422 | 107 |
|  | Dislike | Cold | -7.655 | 39 |
|  | Hand | Warm | -4.542 | 62 |
|  | Hand | Cold | 5.723 | 69 |
|  | Foot | Warm | -4.221 | 39 |
| Water Consumption | Amount of Water | Little | 3.923 | 50 |
|  | Temperature of Water | Warm | 3.134 | 47 |
| Symptoms of Cold | Runny or stuffy nose | No | -3.134 | 47 |
|  | Runny or stuffy nose | Yes | 3.134 | 142 |
|  | Loss of Appetite or Digestion Problems | No | -4.656 | 163 |
|  | Loss of Appetite or Digestion Problems | Yes | 4.656 | 26 |
| In Bad | Digestion Problem | No | -11.48 | 95 |
| Condition | Digestion Problem | Yes | 11.481 | 94 |
