# Supplementary material for: Development of an integrated Sasang constitution diagnosis method using face, body shape, voice, and questionnaire information
Source: BMC Complement Altern Med. 2012 Jul 4;12:85. doi: 10.1186/1472-6882-12-85 (PMC3502327; doi:10.1186/1472-6882-12-85)
Supplement: Additional file 11 — Table S10. Significant binary variables of the questionnaire in SY male patients. [file 1472-6882-12-85-S11.docx]

Table S10. Significant binary variables of the questionnaire in SY male patients

|  | Question | Binary variable  (Answer) | Weight | N |
| --- | --- | --- | --- | --- |
| Personality | Action | Quick | 7.432 | 162 |
|  | Action | Slow | -3.087 | 23 |
|  | Active or Passive | Active | 3.969 | 146 |
|  | Direct or Indirect in Communication | Direct | 3.46 | 129 |
|  | Extrovert or Introvert | Extrovert | 3.923 | 75 |
|  | Extrovert or Introvert | Introvert | -3.087 | 91 |
|  | Energetic or Quiet | Energetic | 6.579 | 134 |
|  | Energetic or Quiet | Quiet | -4.884 | 54 |
| Excrement | Condition | Depending on Food | -3.017 | 85 |
| Cold and Heat | Abdomen | Cold | -3.39 | 29 |
| Headache | Side | No | 3.087 | 218 |
|  | Side | Yes | -3.087 | 24 |
