# Supplementary material for: Development of an integrated Sasang constitution diagnosis method using face, body shape, voice, and questionnaire information
Source: BMC Complement Altern Med. 2012 Jul 4;12:85. doi: 10.1186/1472-6882-12-85 (PMC3502327; doi:10.1186/1472-6882-12-85)
Supplement: Additional file 12 — Table S11. Significant binary variables of the questionnaire in TE female patients. [file 1472-6882-12-85-S12.docx]

Table S11. Significant binary variables of the questionnaire in TE female patients

|  | Question | Binary variable  (Answer) | Weight | N |
| --- | --- | --- | --- | --- |
| Personality | Bold or Delicate | Bold | 4.496 | 134 |
|  | Bold or Delicate | Delicate | -3.576 | 142 |
|  | Masculine or Feminine | Masculine | 4.793 | 159 |
|  | Masculine or Feminine | Feminine | -3.437 | 174 |
| Meal | Meal Size | A lot | 3.599 | 62 |
|  | Eating Speed | Fast | 5.066 | 267 |
|  | Eating Speed | Slow | -3.297 | 69 |
| Digestion | Appetite Sensation | Good | 3.53 | 246 |
|  | Appetite Sensation | Moderate | -4.838 | 159 |
|  | Indigestion | Not at all | 3.946 | 404 |
| Perspiration | Amount | A lot | 8.259 | 151 |
|  | Amount in Hot Weather | A little | -4.129 | 16 |
|  | Feeling after Perspiration | Refreshed | 6.714 | 207 |
|  | Abnormal Perspiration during Eating | No | -3.181 | 417 |
|  | Abnormal Perspiration during Eating | Yes | 3.181 | 79 |
| Excrement | Condition | Depending on Food | -4.267 | 179 |
| Sleep | Duration | >=8 hrs | -3.297 | 60 |
| Cold and Heat | Dislike | Hot | 3.599 | 167 |
|  | Hand | Warm | 10.572 | 186 |
|  | Hand | Cold | -12 | 156 |
|  | Foot | Warm | 5.247 | 113 |
|  | Foot | Cold | -6.016 | 244 |
| Water | Amount of Water | A lot | 5.156 | 125 |
| Consumption | Temperature of Water | Warm | -3.064 | 141 |
| In Bad | Perspiration Problem | No | -6.264 | 421 |
| Condition | Perspiration Problem | Yes | 6.264 | 75 |
|  | Digestion Problem | No | 8.683 | 332 |
|  | Digestion Problem | Yes | -8.683 | 164 |
| Other | Swelling | No | -7.118 | 273 |
| Symptoms | Swelling | Yes | 7.118 | 223 |
