# Supplementary material for: Development of an integrated Sasang constitution diagnosis method using face, body shape, voice, and questionnaire information
Source: BMC Complement Altern Med. 2012 Jul 4;12:85. doi: 10.1186/1472-6882-12-85 (PMC3502327; doi:10.1186/1472-6882-12-85)
Supplement: Additional file 13 — Table S12. Significant binary variables of the questionnaire in SE female patients. [file 1472-6882-12-85-S13.docx]

Table S12. Significant binary variables of the questionnaire in SE female patients

|  | Question | Binary variable  (Answer) | Weight | N |
| --- | --- | --- | --- | --- |
| Personality | Bold or Delicate | Bold | -9.484 | 38 |
|  | Bold or Delicate | Delicate | 7.991 | 181 |
|  | Active or Passive | Active | -7.163 | 140 |
|  | Direct or Indirect in Communication | Direct | -3.761 | 129 |
|  | Direct or Indirect in Communication | Indirect | 3.04 | 156 |
|  | Extrovert or Introvert | Extrovert | -7.185 | 61 |
|  | Extrovert or Introvert | Introvert | 5.881 | 196 |
|  | Energetic or Quiet | Energetic | -8.772 | 109 |
|  | Energetic or Quiet | Quiet | 10.661 | 173 |
|  | Masculine or Feminine | Masculine | -7.32 | 58 |
|  | Masculine or Feminine | Feminine | 12 | 220 |
|  | Opinion Expression | Express well | -4.152 | 128 |
|  | Excited or Calm | Excited | -5.61 | 161 |
|  | Excited or Calm | Moderate | 3.04 | 132 |
| Meal | Meal Size | Excessive | -3.437 | 17 |
|  | Eating Speed | Fast | -6.377 | 135 |
|  | Eating Speed | Slow | 3.111 | 95 |
| Digestion | Good Digestion | Yes | -5.474 | 212 |
|  | Good Digestion | No | 5.474 | 175 |
|  | Discomfort | Null | -5.813 | 209 |
|  | Discomfort | Yes | 3.53 | 141 |
|  | Appetite Sensation | Good | -6.332 | 125 |
|  | Appetite Sensation | Moderate | 4.747 | 189 |
|  | Upset Stomach | Not at all | -3.321 | 217 |
|  | Indigestion | Not at all | -3.04 | 268 |
| Perspiration | Amount | A lot | -5.474 | 52 |
|  | Amount | A little | 3.507 | 174 |
|  | Feeling after Perspiration | Refreshed | -7.723 | 83 |
|  | Feeling after Perspiration | Tired | 5.971 | 166 |
|  | Abnormal Perspiration during Eating | No | 3.669 | 361 |
|  | Abnormal Perspiration during Eating | Yes | -3.669 | 26 |
| Cold and Heat | Dislike | Hot | -4.77 | 75 |
|  | Hand | Warm | -7.073 | 64 |
|  | Hand | Cold | 6.647 | 218 |
|  | Foot | Warm | -4.952 | 37 |
|  | Foot | Cold | 4.336 | 258 |
| Water | Amount of Water | Little | -4.106 | 47 |
| Consumption | Temperature of Water | Warm | 4.929 | 167 |
| In Bad | Digestion Problem | No | -11.64 | 159 |
| Condition | Digestion Problem | Yes | 11.636 | 228 |
| Other | Swelling | No | 3.297 | 277 |
| Symptoms | Swelling | Yes | -3.297 | 110 |
