# Supplementary material for: Development of an integrated Sasang constitution diagnosis method using face, body shape, voice, and questionnaire information
Source: BMC Complement Altern Med. 2012 Jul 4;12:85. doi: 10.1186/1472-6882-12-85 (PMC3502327; doi:10.1186/1472-6882-12-85)
Supplement: Additional file 14 — Table S13. Significant binary variables of the questionnaire in SY female patients. [file 1472-6882-12-85-S14.docx]

Table S13. Significant binary variables of the questionnaire in SY female patients

|  | Question | Binary variable  (Answer) | Weight | N |
| --- | --- | --- | --- | --- |
| Personality | Action | Quick | 3.946 | 296 |
|  | Extrovert or Introvert | Extrovert | 4.404 | 161 |
|  | Extrovert or Introvert | Introvert | -5.836 | 157 |
|  | Energetic or Quiet | Energetic | 3.064 | 232 |
|  | Energetic or Quiet | Quiet | -5.677 | 115 |
|  | Opinion Expression | Express well | 4.175 | 169 |
|  | Opinion Expression | Moderate | -5.903 | 173 |
|  | Opinion Expression | Hide well | -3.576 | 99 |
|  | Careless or Careful | Careless | 3.181 | 148 |
| Cold and Heat | Hand | Cold | 3.087 | 253 |
