# Supplementary material for: Development of an integrated Sasang constitution diagnosis method using face, body shape, voice, and questionnaire information
Source: BMC Complement Altern Med. 2012 Jul 4;12:85. doi: 10.1186/1472-6882-12-85 (PMC3502327; doi:10.1186/1472-6882-12-85)
Supplement: Additional file 15 — Table S14. Selected variables and estimated parameters for face (male). [file 1472-6882-12-85-S15.docx]

Table S14. Selected variables and estimated parameters for face (male)

| SC type |  | B | S.E | Wald | df | p |
| --- | --- | --- | --- | --- | --- | --- |
| SE | Intercept | -0.514 | 0.527 | 0.953 | 1 | 0.329 |
|  | AGE | 0.005 | 0.011 | 0.194 | 1 | 0.659 |
|  | PA(14,21) | 0.249 | 0.247 | 1.022 | 1 | 0.312 |
|  | PDV(14,21) | -0.810 | 0.254 | 10.166 | 1 | <0.001 |
|  | FDH(33,133)/FD(43,143) | 0.340 | 0.172 | 3.887 | 1 | 0.049 |
|  | FD(94,194) | -1.474 | 0.208 | 50.087 | 1 | <0.001 |
|  | FDH(36,136) | 0.329 | 0.173 | 3.613 | 1 | 0.057 |
|  | PDV(6,9) | 0.222 | 0.165 | 1.811 | 1 | 0.178 |
|  | FST(*er*6) | 0.389 | 0.173 | 5.073 | 1 | 0.024 |
| SY | Intercept | -0.688 | 0.479 | 2.064 | 1 | 0.151 |
|  | AGE | 0.018 | 0.010 | 3.190 | 1 | 0.074 |
|  | PA(14,21) | -0.178 | 0.212 | 0.706 | 1 | 0.401 |
|  | PDV(14,21) | -0.253 | 0.203 | 1.548 | 1 | 0.213 |
|  | FDH(33,133)/FD(43,143) | 0.370 | 0.154 | 5.796 | 1 | 0.016 |
|  | FD(94,194) | -0.941 | 0.172 | 29.985 | 1 | <0.001 |
|  | FDH(36,136) | 0.073 | 0.151 | 0.234 | 1 | 0.629 |
|  | PDV(6,9) | 0.417 | 0.136 | 9.356 | 1 | 0.002 |
|  | FST(*er*6) | -0.027 | 0.148 | 0.034 | 1 | 0.853 |

*Model $\chi^{2}=143.2;$ $p<0.0001$, -2 log likelihood=604.8, pseudo $R^{2}$ (Nagelkerke)=0.383

*Reference category: TE type

*B: estimated coefficient, S.E: standard error
