# Supplementary material for: Development of an integrated Sasang constitution diagnosis method using face, body shape, voice, and questionnaire information
Source: BMC Complement Altern Med. 2012 Jul 4;12:85. doi: 10.1186/1472-6882-12-85 (PMC3502327; doi:10.1186/1472-6882-12-85)
Supplement: Additional file 16 — Table S15. Selected variables and estimated parameters for face (female). [file 1472-6882-12-85-S16.docx]

Table S15. Selected variables and estimated parameters for face (female)

| SC type |  | B | S.E | Wald | df | p |
| --- | --- | --- | --- | --- | --- | --- |
| SE | Intercept | 0.990 | 0.437 | 5.130 | 1 | 0.024 |
|  | AGE | -0.030 | 0.009 | 10.473 | 1 | 0.001 |
|  | FST(*er*1) | 0.128 | 0.219 | 0.341 | 1 | 0.559 |
|  | PDV(9,12) | -0.220 | 0.123 | 3.199 | 1 | 0.074 |
|  | FArea03 | -1.429 | 0.650 | 4.841 | 1 | 0.028 |
|  | FA(18,25,43) | 0.105 | 0.157 | 0.446 | 1 | 0.504 |
|  | FD(94,194) | -0.870 | 0.474 | 3.377 | 1 | 0.066 |
|  | FDV(81,50) | 1.681 | 0.519 | 10.484 | 1 | 0.001 |
|  | FDH(33,133) | 0.086 | 0.298 | 0.083 | 1 | 0.773 |
|  | FA(118,117) | 0.353 | 0.132 | 7.148 | 1 | 0.008 |
|  | (FD(18,25)+FD(118,125))/FDH(33,133) | -0.281 | 0.130 | 4.667 | 1 | 0.031 |
|  | PDH(6,7) | 0.273 | 0.151 | 3.283 | 1 | 0.070 |
|  | PDH(12,14) | -0.459 | 0.146 | 9.857 | 1 | 0.002 |
|  | PA(7,6) | -0.023 | 0.155 | 0.022 | 1 | 0.881 |
|  | PA(12,14,21) | -0.250 | 0.141 | 3.152 | 1 | 0.076 |
|  | FCLE*_max_* | 0.094 | 0.145 | 0.425 | 1 | 0.514 |
|  | FDV(*er_max_*, *er*1)/FDH(*er_max_*, *er*1) | -0.159 | 0.205 | 0.597 | 1 | 0.440 |
| SY | Intercept | 1.451 | 0.392 | 13.725 | 1 | <0.001 |
|  | AGE_real | -0.027 | 0.008 | 10.81 | 1 | 0.001 |
|  | FST(*er*1) | 0.584 | 0.186 | 9.877 | 1 | 0.002 |
|  | PDV(9,12) | -0.186 | 0.105 | 3.163 | 1 | 0.075 |
|  | FArea03 | -1.383 | 0.570 | 5.895 | 1 | 0.015 |
|  | FA(18,25,43) | -0.38 | 0.140 | 7.413 | 1 | 0.006 |
|  | FD(94,194) | 0.229 | 0.427 | 0.286 | 1 | 0.593 |
|  | FDV(81,50) | 1.25 | 0.457 | 7.474 | 1 | 0.006 |
|  | FDH(33,133) | -0.511 | 0.274 | 3.472 | 1 | 0.062 |
|  | FA(118,117) | 0.128 | 0.123 | 1.082 | 1 | 0.298 |
|  | (FD(18,25)+FD(118,125))/FDH(33,133) | -0.302 | 0.115 | 6.939 | 1 | 0.008 |
|  | PDH(6,7) | 0.098 | 0.126 | 0.605 | 1 | 0.437 |
|  | PDH(12,14) | -0.371 | 0.126 | 8.663 | 1 | 0.003 |
|  | PA(7,6) | -0.224 | 0.125 | 3.203 | 1 | 0.074 |
|  | PA(12,14,21) | 0.002 | 0.122 | 0 | 1 | 0.988 |
|  | FCLE*_max_* | 0.391 | 0.124 | 9.867 | 1 | 0.002 |
|  | FDV(*er_max_*, *er*1)/FDH(*er_max_*, *er*1) | -0.41 | 0.183 | 5.039 | 1 | 0.025 |

*Model $\chi^{2}=225.3;$ $p<0.0001$, -2 log likelihood=1098.9, pseudo $R^{2}$ (Nagelkerke)=0.349

*Reference category: TE type

*B: estimated coefficient, S.E: standard error
