# Supplementary material for: Development of an integrated Sasang constitution diagnosis method using face, body shape, voice, and questionnaire information
Source: BMC Complement Altern Med. 2012 Jul 4;12:85. doi: 10.1186/1472-6882-12-85 (PMC3502327; doi:10.1186/1472-6882-12-85)
Supplement: Additional file 17 — Table S16. Selected variables and estimated parameters for body shape (male). [file 1472-6882-12-85-S17.docx]

Table S16. Selected variables and estimated parameters for body shape (male)

| SC type |  | B | SE | Wald | df | p |
| --- | --- | --- | --- | --- | --- | --- |
| SE | Intercept | -0.348 | 0.377 | 0.854 | 1 | 0.355 |
|  | AGE | -0.011 | 0.008 | 2.099 | 1 | 0.147 |
|  | WEIGHT | -0.594 | 0.314 | 3.570 | 1 | 0.059 |
|  | BMI | -1.141 | 0.299 | 14.581 | 1 | <0.0001 |
|  | HC/FC | 0.438 | 0.147 | 8.872 | 1 | 0.003 |
|  | WC/NC | -0.337 | 0.168 | 4.046 | 1 | 0.044 |
|  | CC/AC | -0.006 | 0.124 | 0.002 | 1 | 0.960 |
|  | RC/AC | -0.242 | 0.131 | 3.415 | 1 | 0.065 |
|  | NC | -0.931 | 0.213 | 19.062 | 1 | <0.001 |
| SY | Intercept | -0.399 | 0.321 | 1.543 | 1 | 0.214 |
|  | AGE | 0.008 | 0.006 | 1.583 | 1 | 0.208 |
|  | WEIGHT | -0.286 | 0.241 | 1.408 | 1 | 0.235 |
|  | BMI | -0.553 | 0.232 | 5.682 | 1 | 0.017 |
|  | HC/FC | 0.308 | 0.119 | 6.706 | 1 | 0.010 |
|  | WC/NC | -0.390 | 0.14 | 7.75 | 1 | 0.005 |
|  | CC/AC | 0.364 | 0.104 | 12.353 | 1 | <0.001 |
|  | RC/AC | -0.249 | 0.11 | 5.159 | 1 | 0.023 |
|  | NC | -0.674 | 0.175 | 14.793 | 1 | <0.001 |

*Model $\chi^{2}=394.1;$ $p<0.0001$, -2 log likelihood=1304.3, pseudo $R^{2}$ (Nagelkerke)=0.442

*Reference category: TE type

*B: estimated coefficient, S.E: standard error
