# Supplementary material for: Development of an integrated Sasang constitution diagnosis method using face, body shape, voice, and questionnaire information
Source: BMC Complement Altern Med. 2012 Jul 4;12:85. doi: 10.1186/1472-6882-12-85 (PMC3502327; doi:10.1186/1472-6882-12-85)
Supplement: Additional file 18 — Table S17. Selected variables and estimated parameters for body shape (female). [file 1472-6882-12-85-S18.docx]

Table S17. Selected variables and estimated parameters for body shape (female)

| SC type |  | B | SE | Wald | df | p |
| --- | --- | --- | --- | --- | --- | --- |
| SE | Intercept | 0.367 | 0.276 | 1.77 | 1 | 0.183 |
|  | AGE | -0.018 | 0.005 | 10.880 | 1 | <0.001 |
|  | WEIGHT | -0.211 | 0.206 | 1.046 | 1 | 0.306 |
|  | BMI | -1.092 | 0.209 | 27.306 | 1 | <0.001 |
|  | AC | -1.794 | 2.078 | 0.745 | 1 | 0.388 |
|  | CC/AC | -0.908 | 1.101 | 0.681 | 1 | 0.409 |
|  | HC/NC | 0.339 | 0.082 | 17.133 | 1 | <0.001 |
|  | CC | 1.219 | 2.524 | 0.233 | 1 | 0.629 |
| SY | Intercept | 0.940 | 0.238 | 15.602 | 1 | <0.001 |
|  | AGE | -0.014 | 0.005 | 9.246 | 1 | 0.002 |
|  | WEIGHT | -0.514 | 0.173 | 8.853 | 1 | 0.003 |
|  | BMI | -0.242 | 0.169 | 2.044 | 1 | 0.153 |
|  | AC | -6.844 | 1.829 | 13.998 | 1 | <0.001 |
|  | CC/AC | -3.411 | 0.97 | 12.369 | 1 | <0.001 |
|  | HC/NC | 0.127 | 0.069 | 3.345 | 1 | 0.067 |
|  | CC | 7.572 | 2.202 | 11.824 | 1 | <0.001 |

*Model $\chi^{2}=531.251;$ $p<0.001$, -2 log likelihood=2548.9, pseudo $R^{2}$ (Nagelkerke)=0.353

*Reference category: TE type

*B: estimated coefficient, S.E: standard error
