# Supplementary material for: Development of an integrated Sasang constitution diagnosis method using face, body shape, voice, and questionnaire information
Source: BMC Complement Altern Med. 2012 Jul 4;12:85. doi: 10.1186/1472-6882-12-85 (PMC3502327; doi:10.1186/1472-6882-12-85)
Supplement: Additional file 19 — Table S18. Selected variables and estimated parameters for voice (male). [file 1472-6882-12-85-S19.docx]

Table S18. Selected variables and estimated parameters for voice (male)

| SC type | | B | SE | Wald | df | p |
| --- | --- | --- | --- | --- | --- | --- |
| SE | Intercept | 0.525 | 0.408 | 1.656 | 1 | 0.198 |
|  | AGE | -0.035 | 0.008 | 17.554 | 1 | <0.001 |
|  | aENG | -0.143 | 0.335 | 0.183 | 1 | 0.669 |
|  | aMFCC8 | 0.594 | 0.134 | 19.688 | 1 | <0.001 |
|  | aSHIM | 0.179 | 0.106 | 2.846 | 1 | 0.092 |
|  | eMFCC4 | 0.574 | 0.160 | 12.954 | 1 | <0.001 |
|  | eLPR1 | 0.062 | 0.174 | 0.127 | 1 | 0.721 |
|  | iDTF0 | -0.443 | 0.145 | 9.326 | 1 | 0.002 |
|  | iENG | -0.923 | 0.360 | 6.583 | 1 | 0.010 |
|  | iF2/iF1 | -0.448 | 0.151 | 8.859 | 1 | 0.003 |
|  | oDTF0 | 0.179 | 0.197 | 0.830 | 1 | 0.362 |
|  | oMFCC5 | -0.500 | 0.140 | 12.710 | 1 | <0.001 |
|  | oLPR3 | -0.039 | 0.171 | 0.052 | 1 | 0.820 |
|  | sI0 | 0.698 | 0.150 | 21.671 | 1 | <0.001 |
|  | uMFCC2 | 0.478 | 0.142 | 11.351 | 1 | 0.001 |
|  | uMFCC4 | -0.284 | 0.156 | 3.316 | 1 | 0.069 |
| SY | Intercept | -0.506 | 0.391 | 1.673 | 1 | 0.196 |
|  | AGE_real | -0.004 | 0.007 | 0.256 | 1 | 0.613 |
|  | aENG | 0.710 | 0.267 | 7.075 | 1 | 0.008 |
|  | aMFCC8 | 0.242 | 0.116 | 4.322 | 1 | 0.038 |
|  | aSHIM | -0.128 | 0.101 | 1.585 | 1 | 0.208 |
|  | eMFCC4 | -0.267 | 0.145 | 3.378 | 1 | 0.066 |
|  | eLPR1 | 0.505 | 0.160 | 9.900 | 1 | 0.002 |
|  | iDTF0 | -0.509 | 0.141 | 12.988 | 1 | <0.001 |
|  | iENG | -1.344 | 0.375 | 12.857 | 1 | <0.001 |
|  | iF2/iF1 | -0.108 | 0.130 | 0.680 | 1 | 0.410 |
|  | oDTF0 | 0.450 | 0.175 | 6.636 | 1 | 0.010 |
|  | oMFCC5 | -0.135 | 0.121 | 1.245 | 1 | 0.265 |
|  | oLPR3 | -0.385 | 0.146 | 6.971 | 1 | 0.008 |
|  | sI0 | 0.033 | 0.125 | 0.069 | 1 | 0.793 |
|  | uMFCC2 | 0.309 | 0.124 | 6.249 | 1 | 0.012 |
|  | uMFCC4 | -0.325 | 0.126 | 6.683 | 1 | 0.010 |

*Model $\chi^{2}=181.5;$ $p<0.0001$, -2 log likelihood=1047.4, pseudo $R^{2}$ (Nagelkerke)=0.307

*Reference category: TE type

*B: estimated coefficient, S.E: standard error
