# Supplementary material for: Development of an integrated Sasang constitution diagnosis method using face, body shape, voice, and questionnaire information
Source: BMC Complement Altern Med. 2012 Jul 4;12:85. doi: 10.1186/1472-6882-12-85 (PMC3502327; doi:10.1186/1472-6882-12-85)
Supplement: Additional file 20 — Table S19. Selected variables and estimated parameters for voice (female). [file 1472-6882-12-85-S20.docx]

Table S19. Selected variables and estimated parameters for voice (female)

| SC type | | B | SE | Wald | df | p |
| --- | --- | --- | --- | --- | --- | --- |
| SE | Intercept | 0.627 | 0.274 | 5.248 | 1 | 0.022 |
|  | AGE | -0.015 | 0.005 | 7.362 | 1 | 0.007 |
|  | aBW1 | 0.041 | 0.108 | 0.145 | 1 | 0.704 |
|  | aENG | 0.680 | 0.191 | 12.715 | 1 | <0.001 |
|  | aF1 | 0.319 | 0.099 | 10.405 | 1 | 0.001 |
|  | eMFCC3 | -0.076 | 0.097 | 0.609 | 1 | 0.435 |
|  | eSHIM | -0.065 | 0.086 | 0.559 | 1 | 0.455 |
|  | iDTF0 | 0.005 | 0.070 | 0.005 | 1 | 0.942 |
|  | iJITT | -0.135 | 0.140 | 0.932 | 1 | 0.334 |
|  | iPPQ | 0.345 | 0.144 | 5.777 | 1 | 0.016 |
|  | oMFCC5 | -0.143 | 0.091 | 2.469 | 1 | 0.116 |
|  | oPW | -0.349 | 0.163 | 4.595 | 1 | 0.032 |
|  | sLPR3 | 0.445 | 0.130 | 11.644 | 1 | 0.001 |
|  | sF0 | 0.284 | 0.108 | 6.948 | 1 | 0.008 |
|  | uF1 | -0.197 | 0.097 | 4.088 | 1 | 0.043 |
|  | uMFCC2 | 0.098 | 0.100 | 0.950 | 1 | 0.330 |
|  | uMFCC4 | 0.084 | 0.094 | 0.813 | 1 | 0.367 |
| SY | Intercept | 0.946 | 0.256 | 13.655 | 1 | <0.001 |
|  | AGE | -0.018 | 0.005 | 12.140 | 1 | <0.001 |
|  | aBW1 | 0.271 | 0.099 | 7.562 | 1 | 0.006 |
|  | aENG | 0.141 | 0.199 | 0.505 | 1 | 0.478 |
|  | aF1 | 0.231 | 0.093 | 6.212 | 1 | 0.013 |
|  | eMFCC3 | -0.243 | 0.091 | 7.101 | 1 | 0.008 |
|  | eSHIM | -0.198 | 0.085 | 5.415 | 1 | 0.020 |
|  | iDTF0 | 0.263 | 0.090 | 8.455 | 1 | 0.004 |
|  | iJITT | -0.516 | 0.159 | 10.543 | 1 | 0.001 |
|  | iPPQ | 0.625 | 0.150 | 17.468 | 1 | <0.001 |
|  | oMFCC5 | -0.291 | 0.084 | 12.079 | 1 | 0.001 |
|  | oPW | -0.079 | 0.131 | 0.360 | 1 | 0.548 |
|  | sLPR3 | 0.146 | 0.120 | 1.465 | 1 | 0.226 |
|  | sF0 | 0.123 | 0.098 | 1.590 | 1 | 0.207 |
|  | uF1 | -0.085 | 0.088 | 0.924 | 1 | 0.336 |
|  | uMFCC2 | 0.216 | 0.094 | 5.243 | 1 | 0.022 |
|  | uMFCC4 | -0.124 | 0.087 | 2.026 | 1 | 0.155 |

*Model $\chi^{2}=143.0;$ $p<0.0001$, -2 log likelihood=2227.8, pseudo $R^{2}$ (Nagelkerke)=0.136

*Reference category: TE type

*B: estimated coefficient, S.E: standard error
