# Supplementary material for: Development of an integrated Sasang constitution diagnosis method using face, body shape, voice, and questionnaire information
Source: BMC Complement Altern Med. 2012 Jul 4;12:85. doi: 10.1186/1472-6882-12-85 (PMC3502327; doi:10.1186/1472-6882-12-85)
Supplement: Additional file 21 — Table S20. Selected variables and estimated parameters for questionnaire (male). [file 1472-6882-12-85-S21.docx]

Table S20. Selected variables and estimated parameters for questionnaire (male)

| SC type |  | B | SE | Wald | df | p |
| --- | --- | --- | --- | --- | --- | --- |
| SE | Intercept | 0.070 | 0.449 | 0.025 | 1 | 0.876 |
|  | *Q_TE_* | -0.618 | 0.192 | 10.322 | 1 | 0.001 |
|  | *Q_SE_* | 1.030 | 0.218 | 22.316 | 1 | 0.000 |
|  | *Q_SY_* | 0.508 | 0.166 | 9.396 | 1 | 0.002 |
|  | AGE | -0.037 | 0.009 | 16.723 | 1 | 0.000 |
|  | Occupation |  |  |  |  |  |
|  | White collar | 0.577 | 0.291 | 3.929 | 1 | 0.047 |
|  | Blue collar | 0.881 | 0.395 | 4.971 | 1 | 0.026 |
|  | Education |  |  |  |  |  |
|  | <= 9 years | -0.042 | 0.356 | 0.014 | 1 | 0.906 |
| SY | Intercept | 0.075 | 0.396 | 0.035 | 1 | 0.851 |
|  | *Q_TE_* | -0.664 | 0.168 | 15.667 | 1 | 0 |
|  | *Q_SE_* | 0.103 | 0.196 | 0.276 | 1 | 0.599 |
|  | *Q_SY_* | 0.773 | 0.146 | 27.847 | 1 | 0 |
|  | AGE | -0.012 | 0.007 | 2.644 | 1 | 0.104 |
|  | Occupation |  |  |  |  |  |
|  | White collar | 0.017 | 0.237 | 0.005 | 1 | 0.941 |
|  | Blue collar | 0.460 | 0.297 | 2.393 | 1 | 0.122 |
|  | Education |  |  |  |  |  |
|  | <= 9 years | 0.284 | 0.264 | 1.154 | 1 | 0.283 |

*Model $\chi^{2}=291.5;$ $p<0.0001$, -2 log likelihood=1128.7, pseudo $R^{2}$ (Nagelkerke)=0.402

*Reference category: TE type

*B: estimated coefficient, S.E: standard error
