# Supplementary material for: Development of an integrated Sasang constitution diagnosis method using face, body shape, voice, and questionnaire information
Source: BMC Complement Altern Med. 2012 Jul 4;12:85. doi: 10.1186/1472-6882-12-85 (PMC3502327; doi:10.1186/1472-6882-12-85)
Supplement: Additional file 22 — Table S21. Selected variables and estimated parameters for questionnaire (female). [file 1472-6882-12-85-S22.docx]

Table S21. Selected variables and estimated parameters for questionnaire (female)

| SC type |  | B | S.E | Wald | df | p |
| --- | --- | --- | --- | --- | --- | --- |
| SE | Intercept | 0.855 | 0.328 | 6.796 | 1 | 0.009 |
|  | *Q_TE_* | -0.844 | 0.151 | 31.101 | 1 | 0.000 |
|  | *Q_SE_* | 0.466 | 0.170 | 7.490 | 1 | 0.006 |
|  | *Q_SY_* | 0.179 | 0.112 | 2.570 | 1 | 0.109 |
|  | AGE | -0.026 | 0.007 | 13.984 | 1 | 0.000 |
|  | Occupation |  |  |  |  |  |
|  | White collar | -0.128 | 0.188 | 0.466 | 1 | 0.495 |
|  | Blue collar | 0.088 | 0.429 | 0.042 | 1 | 0.838 |
|  | Education |  |  |  |  |  |
|  | <= 9 years | -0.221 | 0.221 | 1.005 | 1 | 0.316 |
| SY | Intercept | 0.795 | 0.298 | 7.116 | 1 | 0.008 |
|  | *Q_TE_* | -0.793 | 0.13 | 37.132 | 1 | 0 |
|  | *Q_SE_* | -0.141 | 0.15 | 0.887 | 1 | 0.346 |
|  | *Q_SY_* | 0.389 | 0.1 | 15.068 | 1 | 0 |
|  | AGE | -0.016 | 0.006 | 6.669 | 1 | 0.01 |
|  | Occupation |  |  |  |  |  |
|  | White collar | 0.299 | 0.163 | 3.351 | 1 | 0.067 |
|  | Blue collar | 0.593 | 0.332 | 3.194 | 1 | 0.074 |
|  | Education |  |  |  |  |  |
|  | <= 9 years | -0.168 | 0.195 | 0.744 | 1 | 0.388 |

*Model $\chi^{2}=346.5;$ $p<0.0001$, -2 log likelihood=2378.0, pseudo $R^{2}$ (Nagelkerke)=0.273

*Reference category: TE type

*B: estimated coefficient, S.E: standard error
